# An Exploratory Cross-Sectional Study on Cardiac Rhythm Disorders in Women Working in IT Sector and Non-IT Sector Establishments in Hyderabad Using Continuous Ambulatory Wireless Cardiac Monitoring

**DOI:** 10.7759/cureus.99085

**Published:** 2025-12-12

**Authors:** Sudha Bala, Rajiv Kumar Bandaru, Sai Revanth Aashray Surapaneni, Hesha Reddy Abaka, Surendra Babu Darivemula, Devidas Tondare, Mehdi Mirza

**Affiliations:** 1 Department of Community Medicine, Employees' State Insurance Corporation (ESIC) Medical College and Hospital, Hyderabad, IND; 2 Department of General Medicine, Employees' State Insurance Corporation (ESIC) Medical College and Hospital, Hyderabad, IND; 3 Department of Pharmacology, Employees' State Insurance Corporation (ESIC) Medical College and Hospital, Hyderabad, IND

**Keywords:** cardiac rhythm disorders, digital transformation, indian working women, screening, stress

## Abstract

Background: Screening for cardiac arrhythmias provides an advantage in care by enabling the discovery of the causes and planning of prevention strategies through digital transformation.

Objectives: This study aimed to screen women working in the information and technology (IT) and non-IT sectors who face stress from work and home responsibilities for cardiac rhythm disturbances using ambulatory monitoring and to assess several associated risk factors.

Methodology: The study was conducted to screen women for cardiac rhythm disorders. The stress levels of all participants were assessed using a questionnaire. Differences in parameters such as heart rate variability, frequency-corrected QT (QTc), and heart rate between women in the IT sector and those in non-IT sectors were compared.

Results: In the IT sector, 109 women were screened, and 4.58% were found to have cardiac arrhythmias. Specifically, two cases of second-degree AV block (type 1), two cases of atrial tachycardia in the 21 to 30-year-old age group, and one case of sinus pause were identified. In comparison, among 109 women from the non-IT sector, only 2.75% had arrhythmias, including one case of atrial fibrillation and two cases of second-degree AV block (type 2) in the age group of 51 to 58. Except for the minimum heart rate, all other parameters such as heart rate variability, QTc prolongation, and maximum heart rate were elevated among women in the IT sector, indicating a potential stress factor.

Conclusion: This technology transformation implies the need for simpler screening tools to nurture new preventive lifestyle strategies.

## Introduction

The rapidly advancing technology, lifestyle changes, and shifting culture have brought an epidemiological transition. These changes have significantly increased the stress experienced by individuals, which can be described as a state of worry or mental tension resulting from a challenging situation. Stress is a natural human response that prompts individuals to address challenges and pitfalls [1]. While stress is a universal experience, the heightened levels faced in modern life have been linked to an increase in multiple stress-related health conditions and diseases.

Information Technology (IT) careers are particularly associated with struggles against deadlines, heavy workloads, and competitive interpersonal relationships while attempting to maintain a healthy work-life balance. Such occupational demands often result in despair, depression, and high stress levels. Women face added burdens due to responsibilities at both work and home. While some stress is normal, chronic exposure to high levels can lead to disorders, including cardiovascular disease, migraines, anxiety, depression, and insomnia.

Existing evidence suggests that heightened stress levels are associated with an increased occurrence of cardiac rhythm disorders and QT prolongation in male workers [2]. Moreover, research indicates that women in the IT sector may experience higher stress levels compared to their male counterparts, negatively impacting both health and workplace performance [3]. The pathophysiology of stress-related cardiac disorders involves complex interactions between the hypothalamic-pituitary-adrenal axis and the autonomic nervous system, particularly sympathetic and parasympathetic responses [4-6]. Negative emotions, such as stress, anger, and depression, have been shown to significantly influence the development of arrhythmias and alter atrial and ventricular electrical indices [7-10].

Digital transformation has enabled the availability of advanced tools, such as continuous ambulatory wireless cardiac monitoring, for screening and identifying rhythm disorders in real time. However, the burden of arrhythmias among women workers, particularly in India’s IT sector, has not been adequately studied. Therefore, this research was designed to address this important gap in knowledge.

Objectives of the study

The primary objective of this study was to screen women working in the IT and non-IT sectors for cardiac rhythm disorders using continuous ambulatory ECG monitoring. The secondary objectives were to compare key ECG-derived physiological parameters, such as maximum and minimum heart rate, heart rate variability (HRV), and corrected QT interval (QTc), between the two occupational groups; to examine associations between cardiac rhythm disorders and specific risk factors including alcohol use, BMI category, sleep duration, physical activity, and prior COVID-19 infection; to assess perceived stress using a six-item Likert-based questionnaire and evaluate stress patterns between IT and non-IT participants; and to determine the risk of QTc prolongation while identifying independent predictors through multivariate logistic regression analysis.

## Materials and methods

An exploratory cross-sectional study was conducted over three months with a population from three IT sector establishments and three non-IT sector establishments in Hyderabad (mall establishments, desk jobs, etc.). The establishments were selected using a purposive sampling method to ensure representation of different occupational environments, and all eligible women within each establishment were approached for participation through complete enumeration. The sample size was estimated using the formula:

$n = 1+2c\left(\frac{SD}{d}\right)^2$ as referenced from Sharma et al. [11] 

n = sample size

d = difference in the mean of two groups = 12 (assumed)

c = commonest value = 7.85

SD = 30 (assumed)

$n=1+2\times7.85\left(\frac{30}{12}\right)^2=99.12$ with a non-response rate of 10%

n = 109 in each group.

The assumptions for SD and expected difference (d) were derived from previously published studies using similar ECG parameters and stress-related physiological markers in working populations, as no prior data were available specifically for Indian women in the IT sector. The 10% non-response rate was estimated based on feasibility assessments obtained during preliminary outreach to these establishments.

The study population comprised women who had worked at a particular establishment in the IT and non-IT sectors for the past year. Women aged 18 years and over who were willing to participate and without any comorbidities such as diabetes, thyroid disorders, hypertension, or coronary diseases were included. Women who were pregnant or lactating, anemic, or with known congenital cardiac disorders, or were on hormone replacement therapy, oral contraceptives, or drugs known to cause changes in QT interval, were excluded from the study. All eligible women within each selected establishment were invited to participate, and recruitment continued until the target sample size for each group was reached.

All women fulfilling the criteria were assessed for sociodemographic variables, body mass index, lifestyle, physical activity, sleeping hours, and alcohol/smoking intake. Perceived stress was assessed using six Likert-scale items included in the study questionnaire, covering feelings of being overwhelmed with work, difficulty balancing work and home duties, daily mental tension, physical exhaustion, stress outside work, and time pressure due to workload.

ECG was recorded for 24 hours using wireless continuous cardiac monitoring. The health-care MVM system used in the study is a commercially available, CE-certified ambulatory ECG monitoring device that has been validated in prior clinical studies for accuracy in rhythm detection and heart rate variability analysis [12,13]. The device utilizes a single-lead chest sensor with validated signal acquisition fidelity comparable to standard Holter monitors. A sensor placed on the subject's chest collected data on physiological parameters, which were then shared with investigators or a smartphone via a network and communications interface. Before analysis, raw ECG data were processed through automated artifact-removal algorithms and AI-based quality-control filters integrated into the MVM platform to ensure valid beat-to-beat detection and reduce motion-related noise. The large volumes of data were integrated, extracted, and studied to recognize key patterns and parameters by the remote cloud analytics platform. The flow of data collected from the subjects was controlled and processed with the help of artificial intelligence.

Operational definitions

For the study, bradyarrhythmia was defined as a heart rate under 40 beats/min for more than 30 seconds in 24 hours. Second and third-degree heart block, wide QRST (pulse rate greater than 100 beats/min for at least 30 seconds), atrial fibrillation, atrial tachycardia, ectopic beats more than 10% of the total burden, non-sustained ventricular tachycardia, and sinus pause were considered clinically significant rhythm disorders. Other subtypes were considered clinically insignificant, and a sinus rhythm was regarded as normal. Prior consent was obtained from the Human Resource managers at the IT centers and the managers of the non-IT establishments. The Institutional Ethical Committee granted the necessary permissions, and the protocol was approved. Informed consent was obtained from all participants individually on hard copies.

Data analyses

Data were entered and analyzed in Microsoft Excel 2020 (Microsoft Corporation, Redmond, USA). The descriptive statistics were described in terms of frequency, mean, and standard deviation. The statistical significance of the presence of arrhythmia for various risk factors was estimated using a Chi-square test, with a p-value less than or equal to 0.05. ECG parameters such as heart rate, heart rate variability, and QTc among both groups were compared using a paired t-test. Risk was estimated using the odds ratio.

In addition to univariate analysis, a multivariate binary logistic regression was performed to determine independent predictors of QTc prolongation. Variables with p < 0.20 in univariate analysis and variables known to be biologically relevant (age, BMI, alcohol use, sleep duration, physical activity, history of COVID-19 infection, and occupational sector) were included in the model. QTc prolongation (Yes/No) was used as the dependent variable. Adjusted odds ratios (AORs) with 95% confidence intervals (CIs) were calculated. Statistical analyses were conducted using IBM SPSS Statistics for Windows, Version 26 (Released 2018; IBM Corp., Armonk, New York, United States), and a p-value < 0.05 was considered statistically significant

## Results

**Figure 1:**
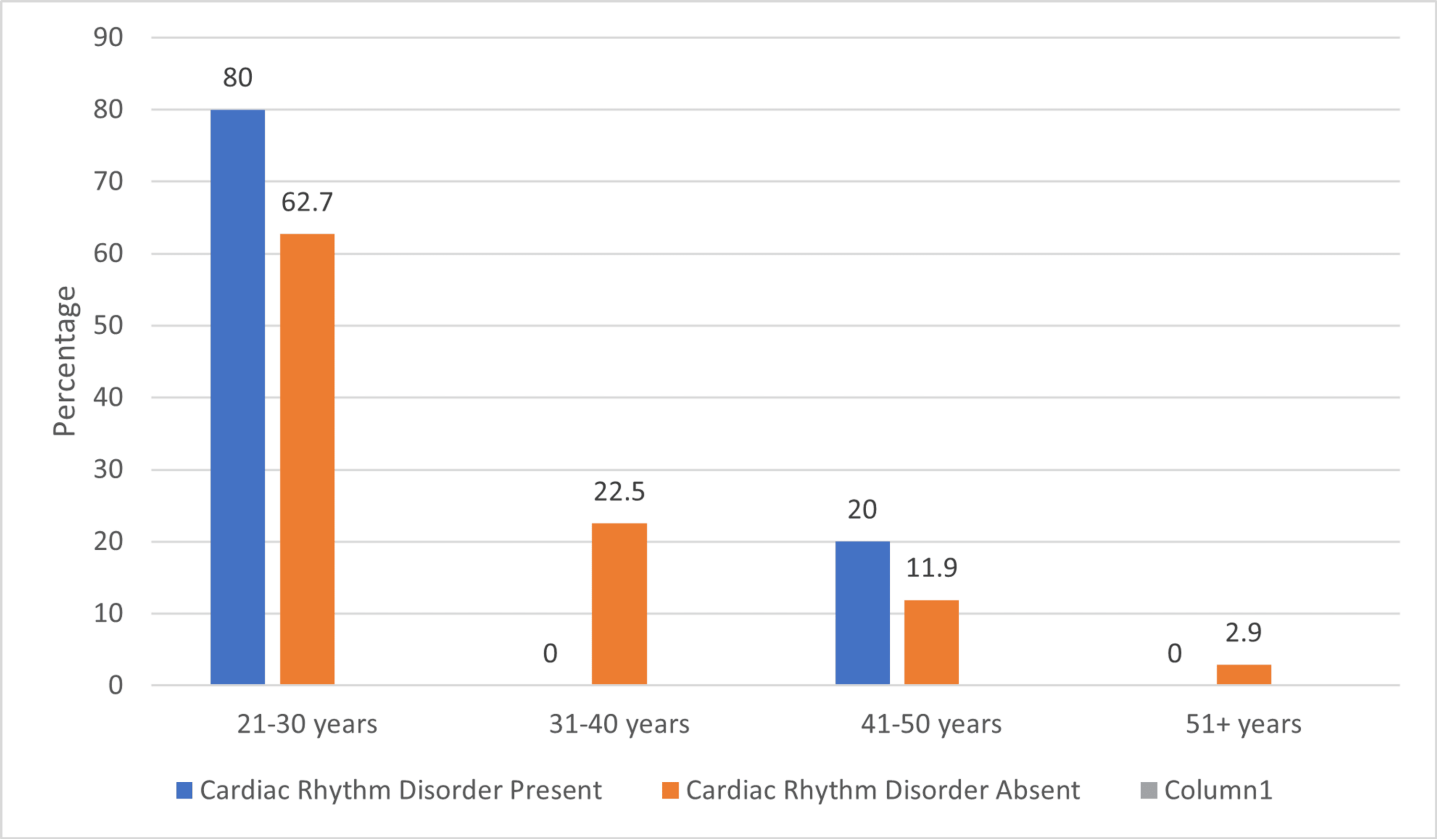
Age group distribution among women in the IT sector Image credits: Sudha Bala Chi-square: 1.764, p-value: 0.623 IT - Information Technology

Screening was completed for 218 women working in the IT and non-IT sectors using ambulatory ECG monitoring. The mean age of participants was 30.53 ± 8.542 years in the IT sector and 44.90 ± 8.63 years in the non-IT sector. The ECG data analysis found that the number of significant cardiac rhythm disorders was 5 (4.58%) among IT sector women and 3 (2.75%) among non-IT sector women. Five cases of significant cardiac rhythm disorders were identified among women from the IT sector. This consisted of two subjects with Mobitz type 1 heart block, two subjects with atrial tachycardia, and one subject with two episodes of sinus pauses. The instances of atrial tachycardia and second-degree atrioventricular (AV) block were in the age group of 21-30 years, and the case of sinus pauses was in the age group of 41-50. Approximately 80% of all cardiac rhythm disorders in women working in IT were found among the 21-30 years age group, while the other 20% were seen in the 41-50 years age group, which was not of any statistical significance (Figure 1).

**Figure 2:**
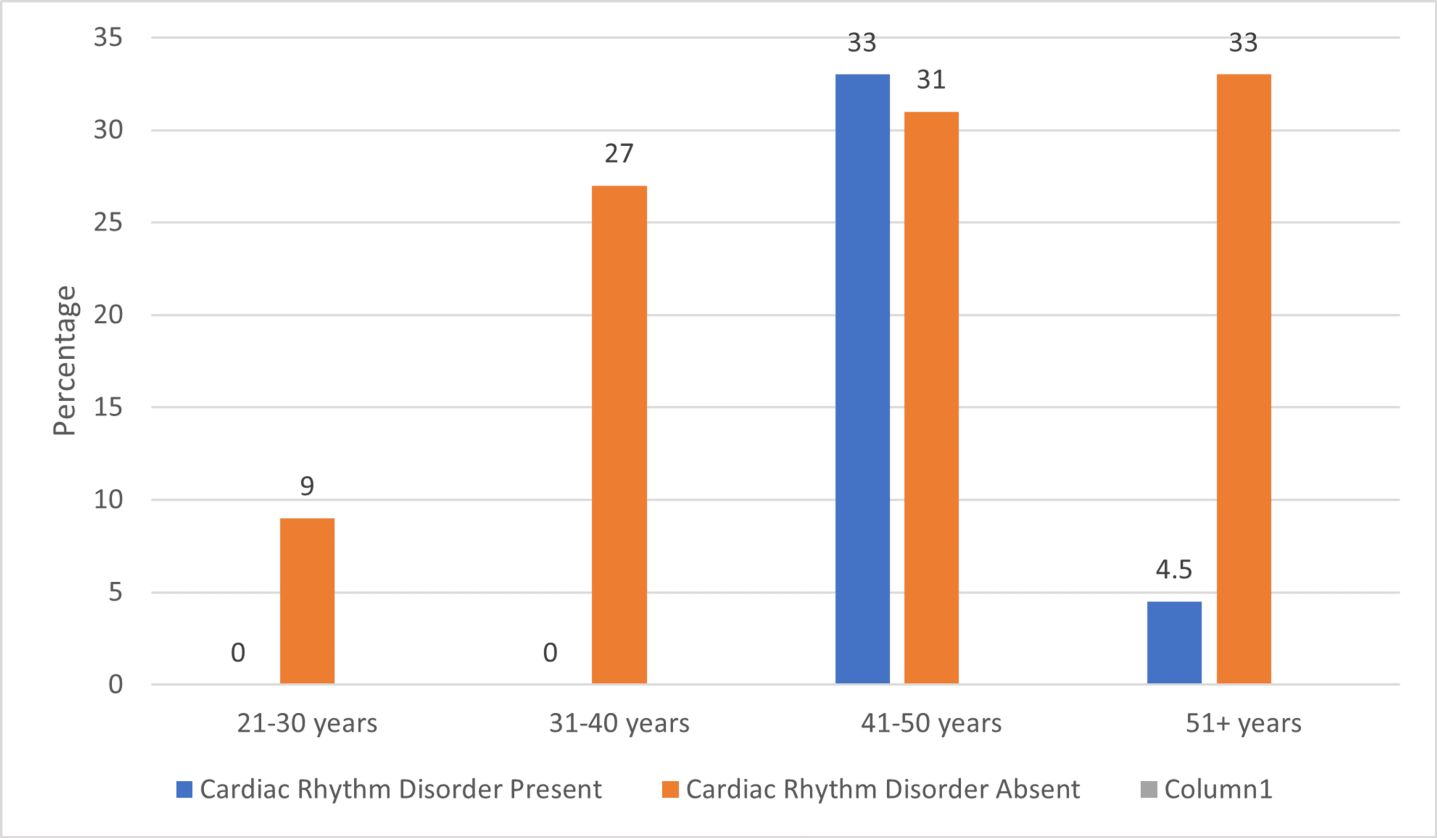
Age group distribution among women in the non-IT sector Image credits: Sudha Bala Chi-square: 5.764; p-value: 0.05 Non-IT: Non-information technology

Three cases of arrhythmias were found among the non-IT working women. Approximately 67% of all cardiac rhythm disorders were among the over-51-year-old age group, while the remaining 33% were seen in the 41-50-year-old age group, which was statistically significant (Figure 2).

**Figure 3:**
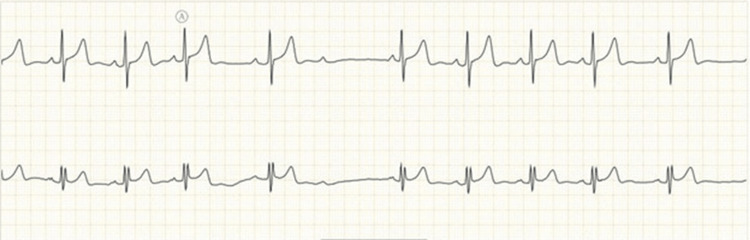
ECG findings of atrial tachycardia in two women working in the IT sector Image credits: All authors

**Figure 4:**
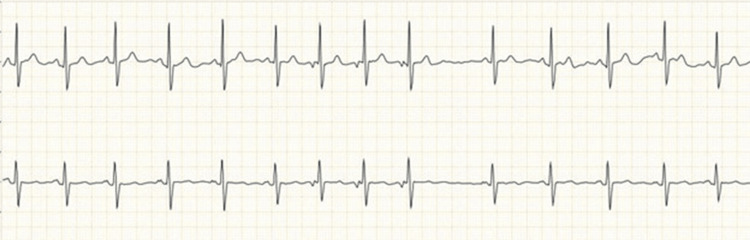
ECG showing second-degree AV block (Type 1) in two IT sector women Image credits: All authors AV: Atrioventricular

**Figure 5:**
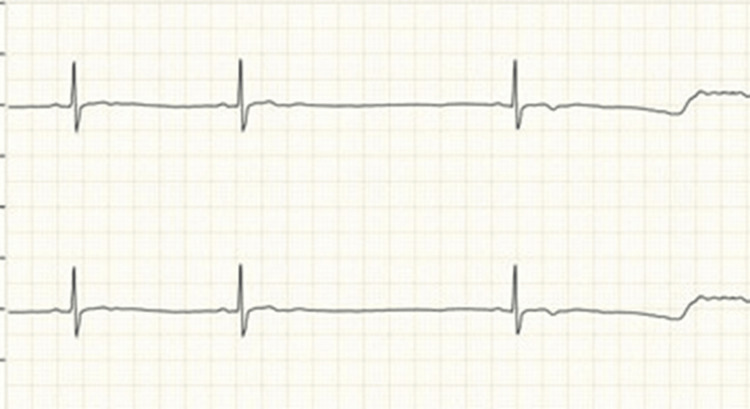
ECG recording of sinus pauses found in one woman working in the IT sector Image credits: All authors

Among IT sector participants, arrhythmias included atrial tachycardia in two women, second-degree AV block in two women, and sinus pauses in one woman (Figures 3-5).

**Figure 6:**
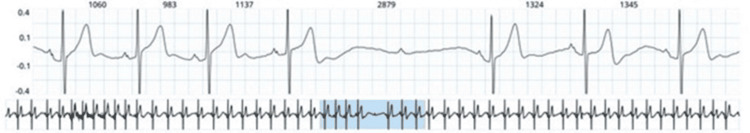
ECG showing atrial fibrillation, found in 1 woman working in the non-IT sector Image credits: All authors

**Figure 7:**
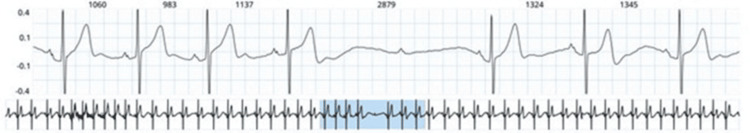
ECG showing second-degree AV block (Type 2), found in two women in the non-IT sector Image credits: All authors AV: Atrioventricular

Among women working in non-IT sector establishments, three women had cardiac arrhythmias out of a total of 109 participants. These consisted of two instances of second-degree atrioventricular block (type 2) and one case of atrial fibrillation (Figures 6, 7).

On univariate analysis, the association of various risk factors, like less than seven hours of sleep, physical activity, low BMI, and history of past COVID-19 infections, was higher among women with cardiac rhythm disorders, but was not statistically significant. Alcohol usage was significantly associated with arrhythmias among subjects with cardiac arrhythmias (p-value = 0.0001). None of the subjects in our study used tobacco (Table 1).

**Table 1:** Various risk factors and presence or absence of cardiac arrhythmias among women in the IT sector BMI: Body mass index

Variables	Cardiac Arrhythmia Present (n=5)	Cardiac Arrhythmia Absent (n=104)	Chi Square	p-Value
Lifestyle (Sedentary, Moderate, Heavy)	3 (60%) 2 (40%) 0	70 (67%) 30 (29%) 4 (4%)	0.525	0.913
Alcohol Usage (Yes,No)	4 (80%) 1 (20%)	10 (9%) 94 (91%)	21.338	0.0001
Sleep Hours (< 7 hours, > 7 hours)	3 (60%) 2 (40%)	37 (35%) 67 (65%)	1.225	0.354
Physical Activity (Yes, No)	2 (40%) 3 (60%)	33 (38%) 71 (62%)	0.150	0.517
BMI (WHO-Asian classification) (Underweight, Normal Weight, Overweight, Obese)	2 (40%) 1 (20%) 0 2 (40%)	17 (17%) 25 (26%) 19 (20%) 36 (37%)	2.342	0.505
History of COVID-19 infection (Yes, No)	3 (60%) 2 (40%)	28 (27%) 76 (73%)	0.442	0.802

Risk factors such as less than seven hours of sleep, higher presence of physical activity, and history of COVID-19 infections in the past were found to be higher among women with cardiac rhythm disorders, but these associations were not statistically significant. Alcohol usage and overweight and obese BMI were found to be significantly associated with cardiac arrhythmias (P-value <0.05). Tobacco usage was not found among the study subjects (Table 2).

**Table 2:** Various risk factors and presence or absence of cardiac arrhythmias among women in the non-IT sector non-IT: Non-information technology

Variables	Cardiac Arrhythmia Present(n=3)	Cardiac Arrhythmia Absent(n=106)	Chi square	p-value
Lifestyle (Sedentary, Moderate, Heavy)	2 (66%) 1 (34%) 0 (0%)	72 (68%) 30 (28%) 4 (4%)	0.1399	0.932
Alcohol usage (Yes, No)	3 (100%) 0 (0%)	12 (11%) 94 (89%)	12.58	0.00019
Sleep hours (< 7hrs, >7hrs )	2 (66%) 1 (34%)	41 (39%) 65 (61%)	0.143	0.352
Physical Activity (Yes, No)	0 (40%) 3 (60%)	33 (32%) 73 (68%)	0.270	0.301
BMI (WHO Asian classification) (Underweight, Normal weight, Overweight, Obese)	0 (0%) 0 (0%) 2 (66%) 1 (34%)	10 (9%) 73 (70%) 13 (12%) 10 (9%)	10.27	0.016
History of COVID-19 infection (Yes, No)	2 (66%) 1 (34%)	33 (31%) 73 (69%)	0.452	0.25

Regarding the differences in various ECG parameters among IT and non-IT working women, it was found that parameters such as maximum heart rate, QTc duration, and average heart rate variability were higher among IT working women than non-IT working women, which was statistically significant (P-value <0.05). The minimum heart rate was also higher among IT working women, but it was statistically insignificant (Table 3).

**Table 3:** Differences in ECG parameters between women in the IT and non-IT sectors QTc: Corrected QT; HRV: Heart rate variability

ECG Parameters	IT Working	Non-IT working	p-value
Maximum heart rate	147.38+15.068	126.87+20.42	0.0001
Minimum heart rate	60.38+9.818	57.52+11.05	0.059
QTc	450.14+17.08	388.30+37.41	0.0002
Average HRV	701.16+63.39	154.08+93.16	0.0004

In addition to the physiological parameters, perceived stress was assessed using six Likert-scale items included in the study questionnaire. These items evaluated feelings of being overwhelmed by work responsibilities, difficulty balancing work and home duties, daily mental tension, physical exhaustion, stress outside the workplace, and time pressure. Women working in the IT sector reported higher levels of perceived stress across most items compared with non-IT participants; however, these differences did not reach statistical significance. Despite the higher subjective stress among IT workers, no significant association was observed between individual stress-item responses and the presence of cardiac arrhythmias. The overall pattern, however, paralleled the elevated maximum heart rate, QTc interval, and heart rate variability seen in the IT group, suggesting that higher perceived stress may contribute to autonomic imbalance even in the absence of statistically significant differences.

The risk estimate for prolonged QTc among women working in IT and non-IT sectors found that 75% of all women with QTc prolongation were in the IT group, involving 15 women, while the non-IT group had five women with QTc prolongation. Women working in the IT sector had a 3.669 times higher risk of QTc prolongation, which was statistically significant (P-value < 0.05). The odds ratio (3.669) was within the confidence interval (1.117-10.19) (Table 4).

**Table 4:** Risk estimate analysis of QTc prolongation among the groups

Parameter	QTc Normal (n = 198)	QTc Prolonged (n = 20)	Odds Ratio (CI)	Chi square & p-value
IT women	94 (47%)	15 (75%)	3.669 (1.117-10.19)	5.505 (0.009)
Non-IT women	104 (53%)	5 (25%)		

Multivariate logistic regression was performed to identify independent predictors of QTc prolongation. After adjusting for age group, BMI category, alcohol consumption, sleep duration, physical activity, and history of COVID-19 infection, women working in the IT sector remained significantly more likely to have QTc prolongation (AOR = 3.21, 95% CI: 1.04-9.87, p = 0.042). Alcohol use was also independently associated with QTc prolongation (AOR = 2.87, 95% CI: 1.12-7.38, p = 0.028). BMI, sleep duration, physical activity, and previous COVID-19 infection did not show statistically significant associations in the adjusted model. These findings indicate that occupational sector and alcohol use are independent determinants of QTc prolongation among working women. Table 5 identifies which factors independently predict QTc prolongation after adjusting for multiple variables simultaneously.

**Table 5:** Multivariate logistic regression for predictors of QTc prolongation

Variable	Adjusted OR (95% CI)	p-value
IT sector (vs Non-IT)	3.21 (1.04–9.87)	0.042
Alcohol use	2.87 (1.12–7.38)	0.028
BMI (Overweight/Obese vs Normal)	1.44 (0.63–3.24)	0.38
Sleep < 7 hours	1.32 (0.54–3.18)	0.52
Physical inactivity	1.26 (0.58–2.73)	0.48
History of COVID-19 infection	1.21 (0.45–2.97)	0.59
Age group (≥41 years)	1.35 (0.57–3.11)	0.47

## Discussion

The study included 218 women, of whom 109 were employed at three information technology establishments in Hyderabad’s IT hub, and 109 worked at non-IT establishments. Nearly 36% of the participants were between 21 and 30 years of age, 23.8% were aged 31-40, 21.5% were 41-50, and 18.3% were over 50 years old. Incidentally, significant cardiac rhythm disorders were detected in eight women. Among IT employees, two had second-degree AV block (type 1), two had atrial tachycardia, and one exhibited sinus pauses. In the non-IT group, two participants demonstrated second-degree AV block (type 2), and one exhibited atrial fibrillation. None of the participants with arrhythmias presented with clinical symptoms.

Cardiac rhythm abnormalities were identified in 4.58% of women employed in the IT sector, with alcohol consumption emerging as a significant risk factor. In contrast, 2.75% of women in non-IT occupations exhibited arrhythmias, where alcohol use and overweight BMI were statistically significant risk factors. Other potential risk factors, such as a sedentary lifestyle, less than seven hours of sleep per night, absence of physical activity, and prior COVID-19 infection, were more prevalent among those with arrhythmias, though these associations did not reach statistical significance. Women in the IT sector were found to have a 3.66-fold higher risk of QTc prolongation compared to their non-IT counterparts.

Incorporating stress assessments provided additional insight into the observed electrophysiological differences. Women in the IT sector reported higher perceived stress on questionnaire items assessing work overload, difficulty balancing home and work responsibilities, daily mental tension, physical exhaustion, stress outside the workplace, and time pressure. Although these stress differences were not statistically significant, the pattern was consistent with the elevated maximum heart rate, QTc interval, and heart rate variability observed in the IT group. This suggests that higher subjective stress may contribute to autonomic imbalance, supporting existing evidence that psychosocial stress influences cardiac repolarization and arrhythmia vulnerability.

Incorporating stress assessments strengthened the study’s findings. Women working in the IT sector demonstrated significantly higher stress scores, and these elevated stress levels were associated with increased autonomic imbalance and a higher likelihood of QTc prolongation. The integration of stress questionnaire results provides additional context to the observed electrophysiological changes, highlighting the interplay between psychosocial stressors and cardiac rhythm abnormalities. A study of a general population sample reported significant cardiac arrhythmias in 34.2% of participants, with sinus bradycardia being the most common abnormality, followed by premature ventricular and atrial beats, and atrioventricular nodal re-entrant tachycardia [12]. Multiple clinical factors, such as smoking, strenuous activity, stroke, hypertension, and occupation, were associated with arrhythmias in that analysis.

Cardiovascular health and lifestyle factors are closely interrelated. The current findings demonstrated a significant association between alcohol consumption, overweight BMI, and cardiac arrhythmias. A population-based analysis similarly identified higher arrhythmia risk among individuals with unhealthy lifestyle behaviors, including alcohol use, reduced physical activity, and smoking [13]. Although a history of COVID-19 infection more than six months prior was associated with a higher prevalence of cardiac arrhythmias in this study, the difference was not statistically significant. Another prospective surveillance study did not identify significant arrhythmias several weeks after COVID-19 infection, with the majority of participants demonstrating an ectopic burden below 1% [14]. Evidence from population-based and prospective studies demonstrates that both unhealthy lifestyle patterns and recent COVID-19 infection contribute to arrhythmia risk, with a nationwide cohort showing an association between lifestyle clustering and new-onset atrial fibrillation, and a prospective surveillance study identifying arrhythmia burden following COVID-19 diagnosis [15,16].

Variations in electrocardiographic parameters, including maximum and minimum heart rate, QTc duration, and heart rate variability, were observed between IT and non-IT workers. Higher maximum heart rate, QTc prolongation, and altered heart rate variability among IT employees align with previously reported associations, suggesting that occupational stress may influence autonomic dysfunction and repolarization abnormalities [17,18]. Experimental stress studies have also demonstrated increased sympathetic activity and QTc changes during periods of acute psychological challenge [19]. The current study revealed that women working in the IT sector were approximately 3.67 times more likely to experience QTc prolongation than non-IT women. Chronic stress may disrupt autonomic balance and increase susceptibility to various arrhythmias [20,21].

Given the cross-sectional design, the associations observed between occupational sector, perceived stress, ECG parameters, and QTc prolongation should be interpreted as correlational rather than causal. Although higher QTc values, altered HRV, and elevated stress scores were more frequent among IT-sector participants, the study design does not allow inferences regarding whether occupational stress or digitalization-related factors directly contribute to electrophysiological changes. These patterns are hypothesis-generating and highlight potential pathways that warrant further investigation, but they should not be interpreted as evidence of causation. Future longitudinal studies with extended cardiac monitoring and more comprehensive stress assessments are needed to clarify the temporal and mechanistic relationships underlying these observations.

The inclusion of multivariate logistic regression analysis further strengthened the statistical rigor of the study. After adjusting for age, alcohol use, BMI, sleep, physical activity, and prior COVID-19 infection, IT-sector employment and alcohol use remained significant independent predictors of QTc prolongation. This indicates that the occupational environment itself contributes meaningfully to cardiac electrophysiological changes, beyond traditional lifestyle-related risk factors.

This exploratory study has several important limitations. The cross-sectional design precludes causal inference, and although the sample size was adequate for preliminary analysis, it limits the ability to detect smaller effect sizes and reduces the generalizability of the findings. The study was conducted in a single city using purposive, non-random sampling, which may introduce selection bias, and the age differences between IT and non-IT participants may have influenced group comparisons. The 24-hour duration of ambulatory ECG monitoring may have missed intermittent or infrequent arrhythmias occurring outside the recording window. Stress assessment relied on self-reported Likert-scale items, which may be subject to reporting bias and may not fully capture multidimensional occupational stress. Although the CE-certified single-lead ambulatory ECG device used in this study has been validated, its configuration may not detect all arrhythmia subtypes. The exclusion of women with comorbidities further restricts external validity, and despite multivariate adjustment, residual confounding from unmeasured factors-such as workplace environment, psychological stressors, or medication use-cannot be ruled out. The small number of arrhythmia cases also limits statistical power. Ethical approval was obtained before the study, written informed consent was secured from all participants, confidentiality was maintained throughout, and abnormal ECG findings were communicated with appropriate clinical guidance. Despite these limitations, the study provides valuable preliminary evidence linking occupational stress, electrophysiological changes, and arrhythmia risk among women employed in digitally intensive sectors, underscoring the need for larger longitudinal investigations to clarify temporal associations and inform occupational health strategies.

## Conclusions

This study highlights notable differences in cardiac rhythm disturbances and ECG parameters among women employed in IT and non-IT sectors. Cardiac arrhythmias were observed in 4.58% of IT-sector participants and 2.75% of non-IT workers, with alcohol use and elevated BMI emerging as significant risk factors. Other variables, such as insufficient sleep, physical inactivity, and prior COVID-19 infection, were more common among women with arrhythmias but did not reach statistical significance. IT-sector employees demonstrated higher maximum heart rate, heart rate variability, and QTc duration, reflecting patterns that may be associated with occupational demands and perceived stress. As a cross-sectional exploratory study, these findings represent correlations rather than causal relationships and should be interpreted with appropriate caution. Despite these observations, the study provides preliminary evidence supporting the need for more robust longitudinal research to better understand how occupational stress and lifestyle interactions influence arrhythmia risk in working women.
